# Disciplinary practices among orphaned children in Sub-Saharan Africa

**DOI:** 10.1371/journal.pone.0246578

**Published:** 2021-02-04

**Authors:** Mark Lee, Elizabeth Heger Boyle

**Affiliations:** Department of Sociology, University of Minnesota, Minneapolis, MN, United States of America; Medical Research Council, SOUTH AFRICA

## Abstract

**Objectives:**

This study considers whether orphans’ experiences with physically and psychologically violent discipline differ from non-orphans in sub-Saharan Africa, and to what extent national, community, household, caretaker, and child characteristics explain those differences.

**Methods:**

We use cross-sectional Multiple Indicator Cluster Surveys (MICS) administered between 2010–2017 in 14 sub-Saharan African countries. The sample included 125,197 children, of which 2,937 were maternal orphans, 9,113 were paternal orphans, and 1,858 were double orphans. We estimate the difference between orphans and non-orphans experience of harsh discipline using multivariable logistic regressions with country fixed effects and clustered standard errors.

**Results:**

Findings show that orphaned children experience *less* harsh discipline in the home. With the exception of double orphans’ experience with physically violent discipline, these differences persisted even after controlling for a rich set of child, household, and caretaker characteristics.

**Conclusions:**

We propose two alternative explanations for our surprising findings and provide a supplementary analysis to help arbitrate between them. The evidence suggests that orphaned children (especially those with a deceased mother) are less likely to experience harsh discipline because of lower caretaker investment in their upbringing. We encourage future research to draw on in-depth interviews or household surveys with discipline data from multiple children in a home to further unpack why orphans tend to experience less harsh punishment than other children.

## 1. Introduction

Attending to the needs of orphans and eradicating harmful forms of discipline have been central aims of international human rights organizations since before the turn of the Millennium. This study considers the intersection of these two important issues by asking whether orphanhood is linked to increases in children’s risk of physically violent or psychologically aggressive punishment.

At the global level, international organizations are unambiguously opposed to harsh discipline. The United Nations Convention on the Rights of the Child (CRC) affirms children’s right to be protected from all forms of physical or psychological harm from parents or caretakers. Bolstering this right, the Committee on the Rights of the Child has issued General Comments stating there is no justification for the use of violent or other cruel or degrading forms of punishment against children [No. 8 (2006) and No. 13 (2011)]. Laws banning corporal punishment, particularly in schools, have spread around the world [1]. Nevertheless, these disciplinary practices continue in high numbers. Nearly 300 million toddlers in the world today (75%) are routinely subject to corporal punishment [2]. UNICEF and other international organizations [3] define orphans as children under 18 years old who have lost one or both parents to death. Although orphans in the world have decreased slightly since 2001, the number of children experiencing orphanhood remains alarmingly high. In 2015, there were nearly 140 million orphans globally, including 52 million in Africa—the focus of this study. About 10.8% of orphans are “double orphans,” meaning both of their parents have died.

Previous research has uncovered orphans’ susceptibility to a number of harms, including lack of food [4], dropping out of school [5, 6], engaging in child labor [7], psychosocial problems [8–11], and increased morbidity [12, 13] and mortality [14, 15]. Female orphans also face an increased risk of sexual abuse, early sexual debut and pregnancy, and sexually transmitted infections [16–18].

To date, few orphan studies using statistical analyses have considered harmful discipline as a primary outcome [19]. Of the few such studies that exist, none controlled for possible confounding factors, such as differences in caretakers. The primary question motivating our study is whether orphaned children are at higher risk of violent physical or psychologically aggressive disciplinary techniques than non-orphaned children in sub-Saharan Africa. We answer this question by using systematic, high-quality survey data to compare orphans’ and non-orphans’ risks of violent and psychologically aggressive discipline while adjusting for confounding sociodemographic factors.

## 2. Correlates of harsh punishment for orphans and non-orphans

The discipline of orphans (and other children) emerges within a complex multileveled system [20, 21]. Community context, and family, caretaker, and child characteristics all play a role in assigning meaning to disciplinary practices, which in turn influences the nature of those practices. Corporal punishment research has uncovered a range of risk factors for harsh discipline; for each set of factors, we consider how orphan status might exacerbate or ameliorate it. Moving from the macro to the micro, we first consider how community norms and household characteristics shape discipline within families; then we review how caretaker and child characteristics, respectively, are related to the occurrence of violent discipline and potentially affect orphans. We consider findings from many prior studies and find a comprehensive analysis of corporal punishment in low- and middle-income countries (LMICs) by UNICEF particularly valuable [22].

### Community characteristics

Marked differences in beliefs about and the practice of harsh discipline around the world suggest that the cultural meaning assigned to these practices varies [23]. This makes the study of aggressive discipline unique from the other harms studied in orphan research, such as dropping out of school or increasing the risk of illness. There is a strong consensus within and across countries that the latter outcomes are harmful. In contrast, the meaning of violent or psychologically aggressive discipline must be interpreted with more caution. In 2015, only five percent of children globally lived in countries that protected children from being hit in their homes [24]. As in many other parts of the world, in most of the countries studied here, a third or more of the caretakers surveyed believe that physical discipline is necessary to “bring up, raise, or educate a child properly.”

Children are more likely to experience violent discipline if caregivers believe such punishment is necessary to properly raise a child [25]. However, large percentages of children receive harsh discipline even if their caregivers believe such punishment is unnecessary. This indicates that caregivers feel pressure to conform to community norms even when those norms go against their personal preferences. To our knowledge, no previous studies have considered the effect of community norms on harsh discipline. The few studies that examine community effects focus on other factors, such as poverty, social cohesion, or collective efficacy [26, 27]. By studying community support for harsh punishment, we will address the significance of the normative context for these parenting behaviors.

Tying this to orphans, the normative context not only helps explain experiences with harsh discipline, but also their meaning. From the perspective of individuals in local communities where harsh discipline is normative, infrequent aggressive discipline could be a sign of indifference or even neglect. It could signal a lack of attention to actions that are considered proper parenting. Discipline, regardless of type, is one form of attention from caregivers, something that most children crave. A common theme from studies of orphans is that orphan caregivers on average invest less time and resources in their young charges [28, 29]. What this means for patterns of discipline is an open question. Adults caring for orphans may feel overwhelmed by their increased responsibilities, leading to impatience with children or less attention to childrearing. In the case of impatience, we would expect to see more corporal punishment [21]. In the case of less attention to children, in communities where corporal punishment is normative, the result could be lower levels harsh discipline (and other types of discipline, as well).

Beyond community norms, we also consider whether a community is urban or rural. The UNICEF study found no consistent relationship for this variable across countries [22]. In those few countries in which household location was statistically-significantly related to harsh discipline, the direction of the relationship varied. Sometimes urban households had more aggressive discipline; sometimes rural households did. Based on the evidence, we have no strong prediction about the effects of residence location on the likelihood of harsh discipline, or whether this variable will mediate the likelihood of harsh discipline for orphans.

### Household characteristics

Previous studies have considered how a range of household factors, including parents’ presence in the home, number of children in the household, and household wealth, influence aggressive discipline. Beginning with parents’ presence in the home, the UNICEF study found no consistent relationship between this and aggressive discipline across countries [22]. There were only three countries in which living apart from parents was associated with greater levels of such discipline; only one (Ghana) was in Africa. This contrasts with other studies showing that families with single parents were more likely to use harsh discipline [21, 30]. To the extent family structure matters, single orphans, especially maternal orphans, are less likely to live with a parent than non-orphans. Double orphans, of course, do not live with their biological parents. All-in-all, the previous research suggests that differences in harsh discipline between orphans, particularly maternal and double-orphans, and non-orphans may be explained by the greater likelihood that orphans do not live with a parent.

Family size is another factor that can affect disciplinary practices. More children can put stress on adult caretakers, leading to more impatience and harsher discipline [21]. The UNICEF study found that, when family size mattered, adults in households with more children were more likely to employ aggressive discipline [22]. Consequently, orphans living in others’ households could increase the risk of aggressive discipline. If couples choose to have large numbers of children, however, orphans in a household would not necessarily increase the risk of aggressive discipline. Notably, the UNICEF study also found that, in most LMICs, family size was not associated with aggressive discipline.

Household wealth and harsh discipline have been studied extensively [26]. Poverty puts extra stress on caretakers, and stress can lead them to a more aggressive approach to childrearing. A meta-analysis of the literature on risk factors for aggressive discipline found a statistically significant relationship between lower socio-economic status and the use of harsh discipline [21]. The UNICEF study focusing on LMICs, in contrast, found that household wealth (measured dichotomously) was not related to the use of such discipline in most countries [22]. In countries where there was a relationship, poorer households were more likely to report the use of aggressive discipline. The discrepancy may be explained in differences in the distribution of poverty within countries. In LMICs, rural areas tend to have high levels of poverty, but also high levels of social cohesion. In the global north, poverty is more likely to be associated with urban areas and high levels of social disorganization [21].

In many locales, orphans are more likely to live in poor households. The loss of an adult family member can lead to a decrease in a household’s standard of living [10]. As an illustration, one study discovered that orphans were uniquely vulnerable to dropping out of school, food insecurity, and child labor, harms which signal reduced household resources [7]. To the extent poorer households engage in harsher discipline, our results may show an increased risk of this type of discipline for orphans.

Household characteristics are not distributed randomly across non-orphans and different types of orphans. To the extent different categories of children are at varying risks of aggressive discipline, differences in households may explain some of that variation. In our models, we control for whether the head of the household is the child’s parent, the number of children in each household, wealth, and urban residence.

### Caregivers

For orphans, caregivers are an important mediating factor between orphan status and a range of deleterious effects. As noted above, caregivers typically invest less time and resources in orphans relative to their own biological children. This may lead to worse outcomes for double-orphans versus orphans with a surviving parent. Caregiver attitudes toward harsh discipline also influence their use of these tactics [25]. It is possible that individuals willing to take in orphans have more childrearing experience, with attitudes toward violent discipline that vary from other caregivers.

The gender, age, education, and mental health of caregivers are sometimes related to the decision to use aggressive discipline. Some studies have found women are more likely to engage in aggressive childrearing practices than men, perhaps because societal gender hierarchies lead children to test women caregivers’ authority more than men’s [31]. Maternal orphans are more likely to have men as caregivers, while paternal orphans are more likely to have women. This could explain variation in the likelihood of aggressive discipline for these two groups. Greater caregiver education appears to reduce children’s risk of aggressive discipline [22], and this is independent of education’s association with socio-economic status [32]. There is not a clear basis for expecting orphan caregivers to have more or less education than the caregivers of non-orphans, once household socio-economic status is controlled.

With respect to age, some studies have found that younger caregivers are more likely to use harsh disciplinary practices [21, 30]. Younger caretakers may have their authority challenged more; or older caregivers may eschew violent punishment because it is physically taxing or because they have developed childrearing skills that allow them to avoid it. Orphans are, on average, older than other children since orphanhood occurs after birth. Consequently, their caretakers are also older on average, which could suggest lower levels of aggressive discipline.

Caregivers of orphans are more likely to report poor psychological health than caregivers of non-orphans. Like the orphans they care for, they have often experienced the loss of a loved one, and may be going through a bereavement process [33]. They may also be dealing with stressful changes in their household structures. Although we cannot model caregiver mental health specifically in our statistical analyses, it provides additional reason to expect that orphans will be more likely to experience harsh disciplinary practices.

### Child characteristics

Certain children are more susceptible to aggressive discipline than others, even after controlling for household and caregiver factors. One study found that boys were slightly more likely than girls to experience harsh discipline [31], but other studies have not uncovered an effect for gender [22]. To the extent that discipline is an investment in a child by a caregiver, the research on orphans suggest female orphans receive more attention than male orphans. This is not entirely a good thing, however, as female orphans are more likely to experience sexual abuse [18]. As explained above, more attention may also translate into more harsh discipline if community norms and caregiver attitudes support corporal punishment.

A child’s age is one of the most important predictors of harsh punishment. Children 5 to 9 years old are more likely to experience harsh discipline than older or younger children [22]. As we noted above, orphans on average are older than other children, and this may be a significant factor in their risk of experiencing aggressive discipline.

Children with behavioral problems tend to experience more aggressive discipline [34]. Orphans tend to have higher levels of psychosocial stress than other children because of the experience of losing a parent, and this can contribute to greater behavioral problems [8]. Research shows that children who lose a mother are especially vulnerable to this harm. In terms of the implications for violent discipline, children who are experiencing psychosocial distress may be more likely to misbehave, leading to greater levels of violent discipline. To the extent orphans’ exposure to harsh discipline is significantly different from other children’s even after controlling for the myriad factors identified here, this could be one explanation. The previous research also suggests that maternal orphans may be at greater risk of aggressive discipline than other orphans.

## 3. Methods

### 3.1 Data

Data for this cross-sectional study come from the Multiple Indicator Cluster Surveys (MICS), which are household surveys developed by UNICEF in partnership with administering countries. The MICS sampling strategy involves randomly selecting one child, aged from 2 to 14, from the household roster for the disciplinary-practices module. That child’s primary caregiver answers the relevant questions. Because the surveys select from populations of children rather than mothers, MICS are unique among household surveys for including orphans within their sampling frame.

The number of orphans within each country sample tends to be very small, thus limiting statistical power to detect significant differences. We pool data from 14 sub-Saharan African countries to allow a robust multivariable analysis. Specifically, these data come from the most recent available MICS from Benin, Cameroon, the Central African Republic, Chad, Cote d’Ivoire, Eswatini (formerly Swaziland), Ghana, Malawi, Mali, Mauritania, Nigeria, Sierra Leone, Somalia, and Zimbabwe. Other sub-Saharan African countries did not have data available at the time of our analyses. The data from Somalia come from two sub-national surveys, one focused in the Northeast Zone and the other in Somaliland (before this portion of Somalia became an independent country). All surveys were conducted between 2010 and 2017. The pooled sample included 134,548 children aged 2–14. Approximately 5 percent (N = 6,482) of this sample was excluded due to missing data on child discipline. Additionally, 2 percent of the sample (N = 2,869) were excluded due to missing data on one or more covariates. The final sample includes 125,197 children.

### 3.2 Variables

Our dependent variable comes from the MICS child discipline module. MICS include a series of questions on violent and psychologically aggressive discipline. These questions, selected by a panel of international experts and field tested in low to middle income countries [35, 36], were adapted from the Parent-Child Conflict Tactics Scale [37] and the WorldSAFE survey [38]. The S1 Table provides a list of the items that comprise violent discipline and psychologically aggressive discipline from the MICS. Violent discipline includes shaking, spanking, slapping, hitting with a hand or object, and beating. Psychologically aggressive discipline includes shouting, yelling, screaming, and name-calling to degrade or humiliate the child. Throughout this article, we use the terms “harsh” or “aggressive” discipline to encompass both physically violent and psychologically aggressive discipline. Children’s caretakers report whether 11 different disciplinary actions had been used with the children in the last 30 days by any household member. We constructed dichotomous indicators of physical violence and psychological aggression, where the presence of any form of punishment in each category is coded as one. The full question wording and classification are available in the S1 Table.

Our main independent variable, orphan status, is a 4-category variable that indicates whether a child is a non-orphan, maternal orphan (mother only deceased), paternal orphan (father only deceased), or double orphan (both parents deceased). These categories are mutually exclusive. This variable is constructed from separate questions on the household roster that asks for all children under age 18 whether their mother and father are living.

Orphaned and non-orphaned children differ from each other systematically in important ways. To reduce bias in our statistical models, we adjust for a rich set of covariates measured at multiple levels. MICS surveys report child’s age, gender (male or female), and relationship to the household head (biological child, non-child relative, or non-relative). Characteristics of the child’s primary caretaker are also measured, including age, gender, education level (none/primary, secondary, post-secondary, or religious), and whether he or she believes that corporal punishment is necessary to properly bring up a child. At the household level, we include wealth (top 40 percent versus bottom 60 percent) and the number of children under 17. We also include two community-level variables. The first is an indicator or urban versus rural. The second indicates the proportion of households in the survey cluster (a rough proxy for community) in which a caretaker indicated support for corporal punishment.

### 3.3 Analytic approach

Disciplinary differences between orphans and non-orphans in sub-Saharan Africa may result from between-country differences and within-country differences. A between-country difference would be most prominent if a large portion of the orphan population resided in countries where aggressive discipline was either more or less common than countries with fewer orphans. A within-country difference would result from a large difference between orphaned and non-orphaned children in each country. We first conduct simple descriptive analyses showing how both between- and within-country differences contribute to disciplinary differences between orphans and non-orphans in our pooled sample.

Observed differences between orphans and non-orphans could also be an artefact of child, caretaker, household, and community differences as described above. The number of orphans in any single survey is too small to allow a robust multivariable analysis. We use the pooled sample to increase statistical power. First, we describe differences in our sample across categorical and continuous control variables using chi-squared or ANOVA to test for significance. Then, we use logistic regression equations with country fixed effect to model the log odds of experiencing each punishment type as a function of orphan status. Next, we add covariates to estimate whether orphan status predicts disciplinary experiences independently of these differences. The amount of missing data was negligible, so our models use listwise deletion to retain only complete cases.

Analyses were conducted using Stata 15.1. We used the *svy* command in Stata to account for the complex survey design, which includes multistage stratified cluster sampling. The MICS data include a household weight to account for unequal probability of selection and unit non-response. Since only one child in each household is selected for the child discipline module, children from larger families have a lower probability of being included. We account for this by multiplying the household weight by the number of children aged 2–14 in the household. The resulting child weight is used in all analyses.

This study was exempt from review by an institutional review board because it relied solely on publicly available, non-identifiable secondary data analysis.

## 4. Results

### 4.1 Between-country differences

**Fig 1:**
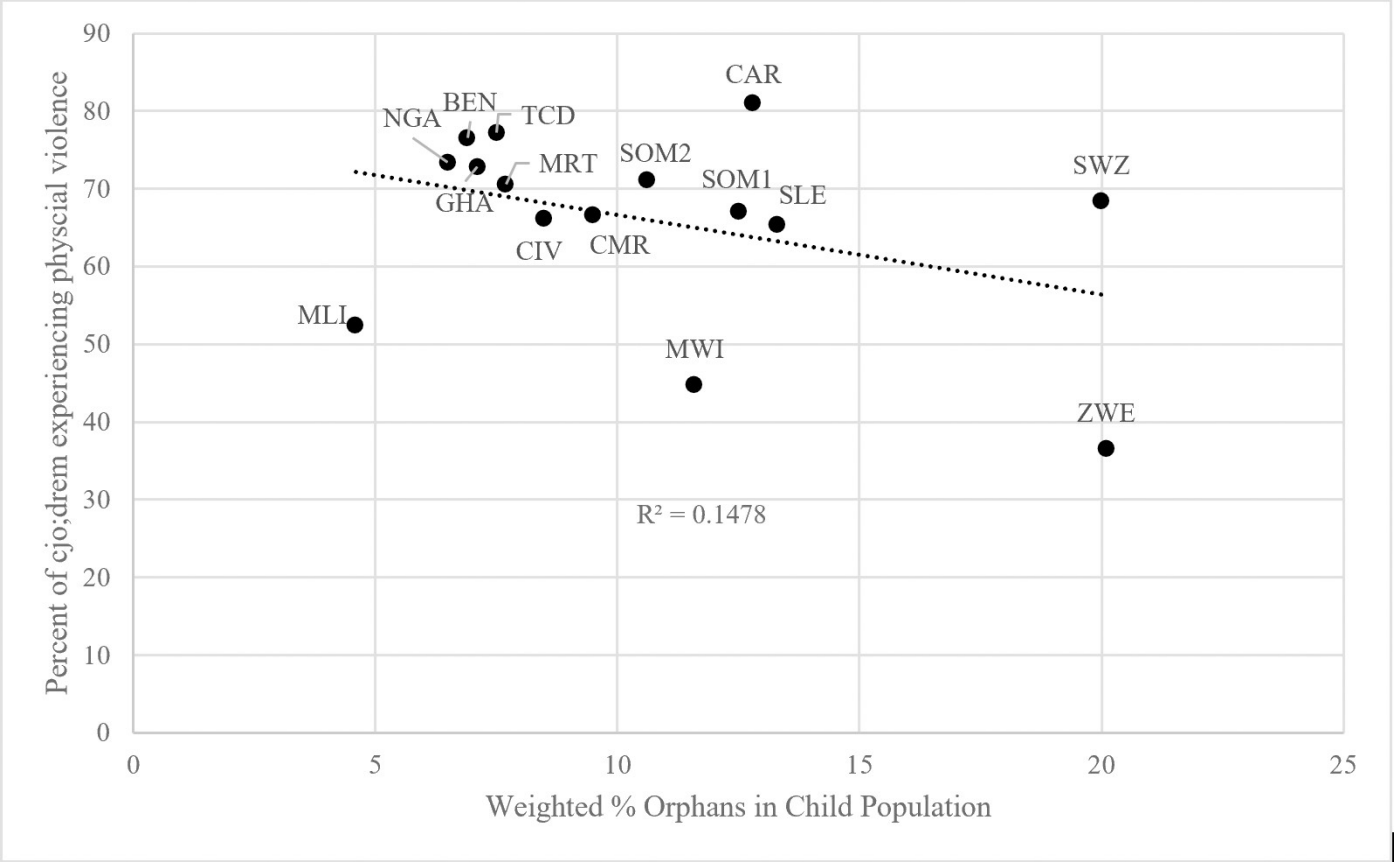
Relationship between physical violence and percentage of orphans in population. BEN = Benin, CMR = Cameroon, CAR = Central African Republic, TCD = Chad, CIV = Cote d’Ivoire, GHA = Ghana, MWI = Malawi, MLI = Mali, MRT = Mauritania, NGA = Nigeria, SLE = Sierra Leone, SOM1 = Somalia (Northeast Zone), SOM2 = Somalia (Somaliland), SWZ = Swaziland, ZWE = Zimbabwe.

Neither orphans nor harsh discipline are evenly distributed across the sub-Saharan African countries in our sample. As shown in Table 1, the percentage of orphans varies across countries. As a weighted average, 90.1 percent (N = 111,289) of the pooled sample are non-orphans, 2.1 percent (N = 2,937) are maternal orphans, 6.5% (N = 9,113) are paternal orphans, and 1.3% (N = 1,858) are double orphans. If the percentage of orphans in a country varies with the commonness of aggressive discipline, this could explain some of the disciplinary differences between orphans and non-orphans. Fig 1 plots the percent of orphans (for simplicity, defined as children with at least one deceased parent) in the child population and the prevalence of physically violent punishment in the home. The trend line shows a slight negative relationship (R^2^ = 0.148). Zimbabwe has the highest percentage of orphans (20.1%) and the lowest levels of physical violence (36.5%). Meanwhile, countries with some of the lowest levels of orphanhood, like Benin (6.9%) and Nigeria (6.5%), report some of the highest levels of physical violence (76.6% and 73.4% respectively). By and large, orphans are somewhat less likely to live in countries where physically violent discipline is common.

**Table 1:** Description of sample countries.

Country	Number of Children	Orphan Prevalence				Discipline Prevalence		
		Non-orphan	Maternal	Paternal	Double	Physical Violence	Psychological Aggression	Support for Physical Punishment
Benin	8,680	93.1	2.1	4.3	0.5	76.6	89.4	46.6
Cameroon	5,389	90.5	2.1	6.6	0.8	66.6	82.0	47.1
Central African Republic	8,065	87.2	3.2	7.9	1.7	80.9	84.4	31.3
Chad	11,722	92.5	1.6	5.2	0.7	77.2	71.5	41.6
Cote d'Ivoire	6,691	91.5	2.6	5.3	0.7	66.2	84.9	31.6
Ghana	8,065	92.9	1.6	4.6	0.9	72.8	88.6	51.6
Malawi	17,502	88.4	2.0	7.4	2.1	44.7	68.9	6.1
Mali	2,195	95.4	0.8	2.4	1.4	52.3	65.2	34.5
Mauritania	7,575	92.3	1.8	5.4	0.6	70.6	74.3	50.9
Nigeria	19,302	93.5	1.8	4.2	0.6	73.4	77.6	63.5
Sierra Leone	9,016	86.7	3.3	7.9	2.0	65.5	75.0	44.1
Somalia (Northeast Zone)	3,875	87.5	1.4	10.1	1.0	67.1	67.0	36.8
Somalia (Somaliland)	3,909	89.4	1.8	7.8	1.0	71.2	68.0	31.5
Swaziland	2,837	80.0	4.2	13.2	2.7	68.4	81.8	66.3
Zimbabwe	10,374	79.9	2.7	13.5	3.9	36.5	54.6	38.4
Total	125,197	90.1	2.1	6.5	1.3	66.0	75.3	41.4

**Fig 2:**
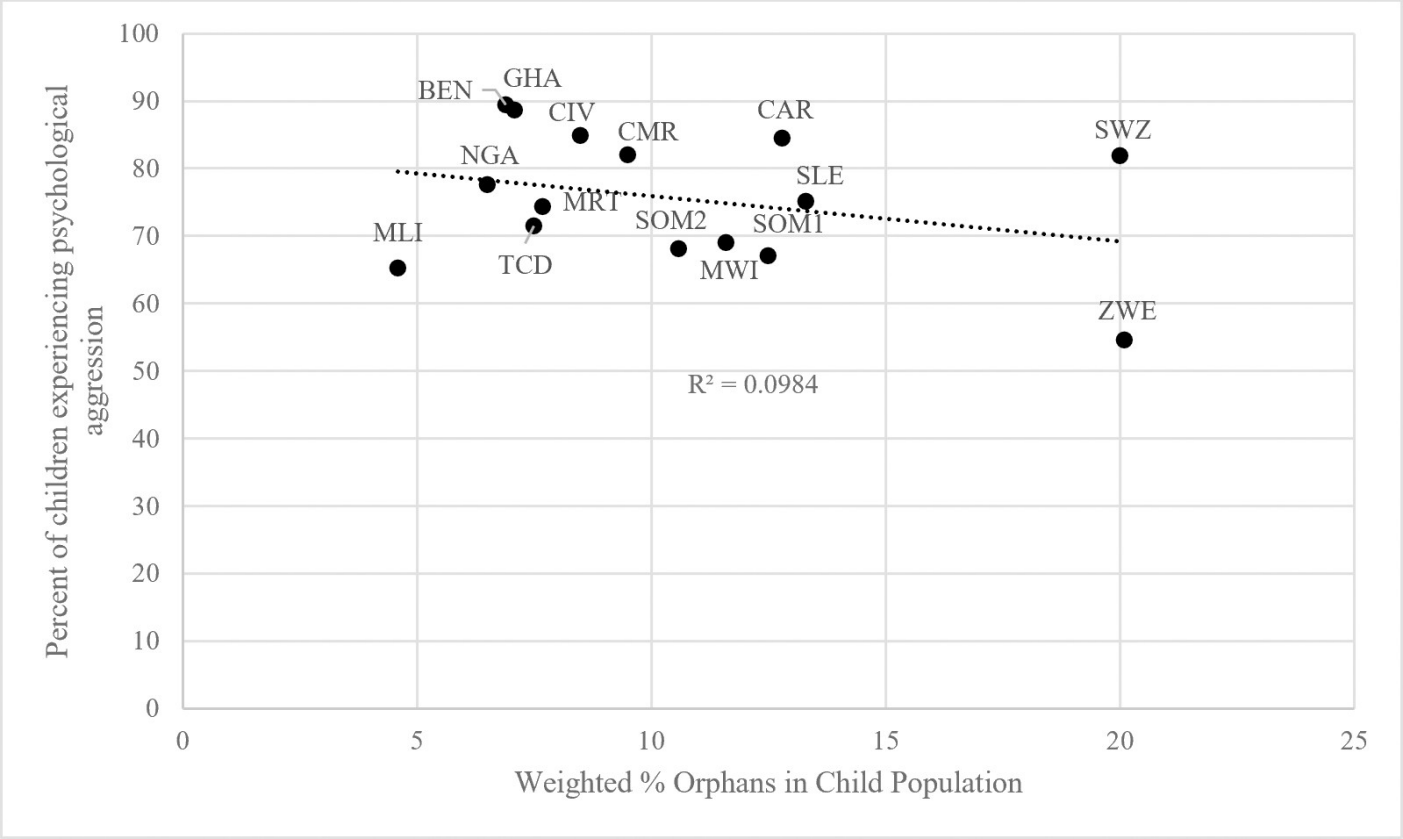
Relationship between psychological aggression and percentage of orphans in population. BEN = Benin, CMR = Cameroon, CAR = Central African Republic, TCD = Chad, CIV = Cote d’Ivoire, GHA = Ghana, MWI = Malawi, MLI = Mali, MRT = Mauritania, NGA = Nigeria, SLE = Sierra Leone, SOM1 = Somalia (Northeast Zone), SOM2 = Somalia (Somaliland), SWZ = Swaziland, ZWE = Zimbabwe.

The same is true for psychologically aggressive discipline, presented in Fig 2. Again, Zimbabwe has more orphaned children as a percentage of its population than other countries and reports the lowest prevalence of psychological aggression (54.6%). In contrast, Benin has a relatively small orphan population, but psychological aggression there is nearly a universal tactic (89.4%). Aggressive discipline is generally less common in countries where orphans constitute a greater share of the child population.

It is unclear why these variables relate to each other in this way across the sample countries, and we make no causal claim that one effects the other at the national level. Because orphans tend to live in countries where harsh discipline is used less frequently in this region of the world, however, it is important that our models include country fixed effects.

### 4.2 Within-country differences

**Fig 3:**
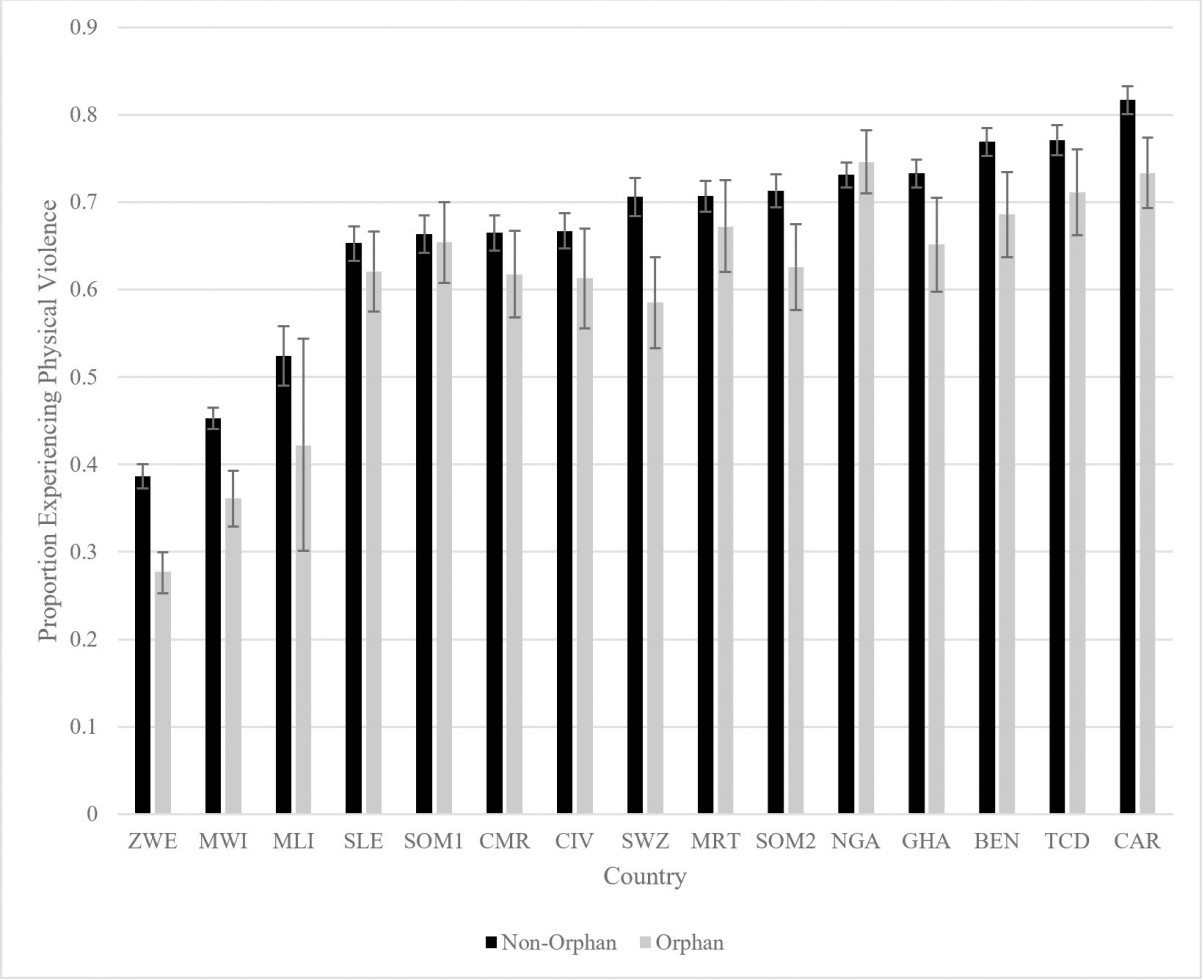
Physical violence by orphan status within sample countries. BEN = Benin, CMR = Cameroon, CAR = Central African Republic, TCD = Chad, CIV = Cote d’Ivoire, GHA = Ghana, MWI = Malawi, MLI = Mali, MRT = Mauritania, NGA = Nigeria, SLE = Sierra Leone, SOM1 = Somalia (Northeast Zone), SOM2 = Somalia (Somaliland), SWZ = Swaziland, ZWE = Zimbabwe.

In addition to between-country differences, we examine disciplinary differences between all orphans (combined) and non-orphans within each country. Fig 3 shows the bivariate relationship between orphan status and physically violent punishment. Orphans are less likely to experience physical violence in the home in 14 out of 15 samples. In the one survey where orphans are more likely to experience physical violence, the differences are small and insignificant. Differences are statistically significant (p<0.05) in 12 of the surveys where physical violence is reportedly less common among orphaned children. The country with the largest difference is Zimbabwe, where 38.6 percent of non-orphans are physically punished compared with 27.7 percent of orphans. Overall, orphans tend to be at lower risk of physical violence than non-orphans within most countries in our study.

**Fig 4:**
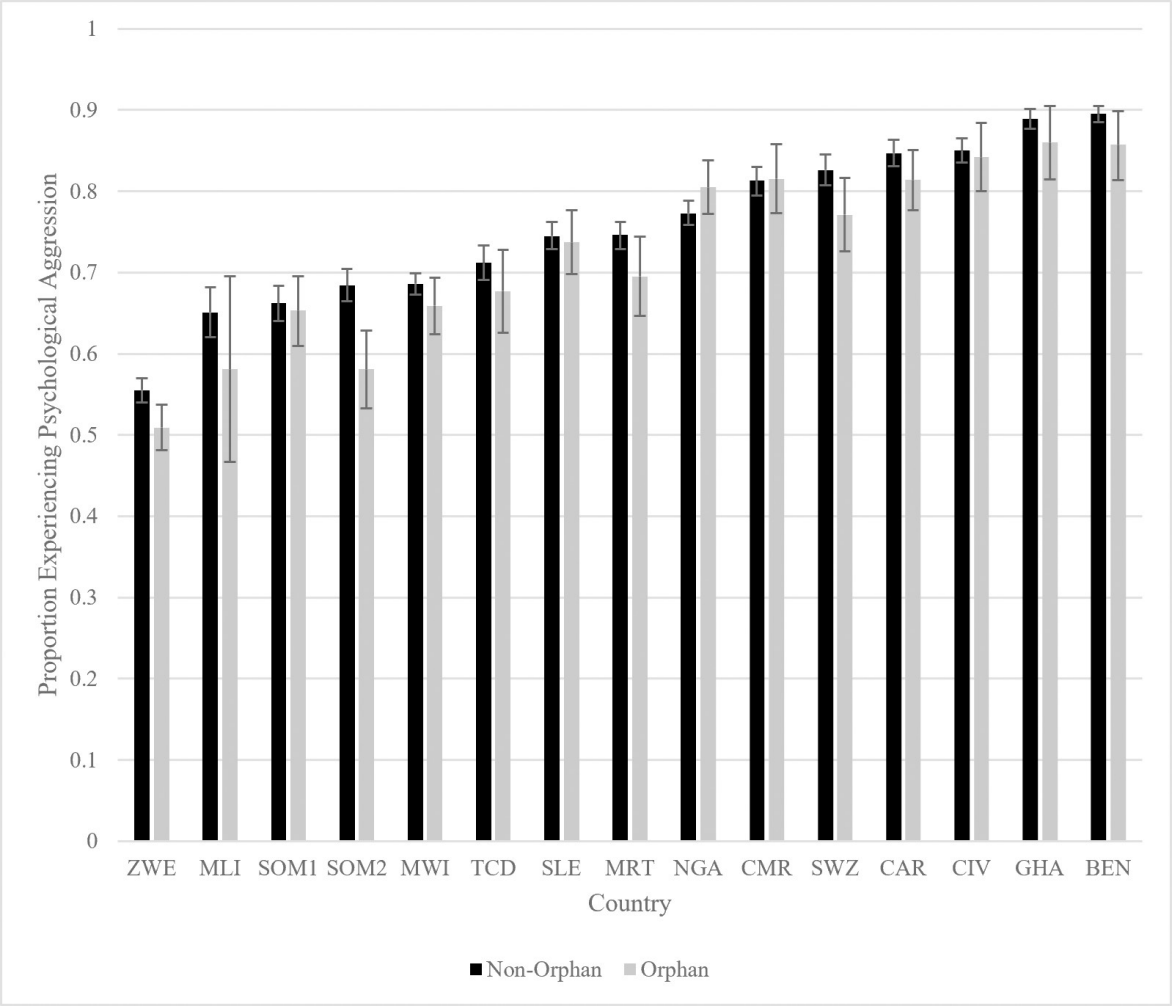
Psychological aggression by orpan status within sample countries. BEN = Benin, CMR = Cameroon, CAR = Central African Republic, TCD = Chad, CIV = Cote d’Ivoire, GHA = Ghana, MWI = Malawi, MLI = Mali, MRT = Mauritania, NGA = Nigeria, SLE = Sierra Leone, SOM1 = Somalia (Northeast Zone), SOM2 = Somalia (Somaliland), SWZ = Swaziland, ZWE = Zimbabwe.

A similar trend emerges when analyzing psychologically aggressive discipline. Fig 4 shows these results. In 13 out of 15 samples, orphans are less likely to experience psychological aggression than non-orphans, but this difference is statistically significant (p<0.05) in only two of these countries. In total, orphans tend to experience slightly lower levels of psychological aggression than non-orphans within each country, although the differences are smaller and less robust than for physical violence.

### 4.3 Pooled multivariable analyses

The above descriptive results suggest that orphans are at lower risk of experiencing aggressive discipline in the home. To unpack the meaning of the bivariate results, it is essential to consider if differences persist across orphan types (i.e. maternal, paternal, and double) or whether the extent to which orphan/non-orphan differences are explained by variation between orphans and non-orphans in child, caretaker, household, and community characteristics. We do this by conducting robust multivariable analyses.

Table 2 provides a description of the pooled sample. For dichotomous and categorical variables, we performed a chi-squared test to assess whether there were significant differences across orphan statuses. We conducted an ANOVA test for the continuous variables. P-values from these tests are reported in the far-right column of the table. As expected from the single-country analyses, orphans in the pooled sample are significantly less likely to experience harsh discipline. Among non-orphans, 66.9% experience physically violent discipline and 75.7% experience psychologically aggressive discipline. The numbers are lower for maternal orphans (57.3% PVD and 70.1% PAD), paternal orphans (59.1% PVD and 72.9% PAD), and double orphans (49.5% PVD and 64.5% PAD).

**Table 2:** Description of pooled sample.

Variable	Total (N = 125,197)	Non-Orphans (N = 111,289)	Maternal Orphans (N = 2,937)	Paternal Orphans (N = 9,113)	Double Orphans (N = 1,858)	p
**Discipline Type**						
Physical Violence	66.0	66.9	57.3	59.1	49.5	<0.001
Psychological Aggression	75.3	75.7	70.1	72.9	64.5	<0.001
**Child**						
Age	7.6 (0.02)	7.4 (0.02)	9.1 (0.09)	9.1 (0.06)	10.0 (0.11)	<0.001
Sex (Female)	50.0	49.9	48.5	51.3	52.9	0.071
**Caretaker**						
Age	37.0 (0.05)	36.2 (0.05)	44.8 (0.41)	42.7 (0.19)	49.1 (0.61)	<0.001
Sex (Female)	97.2	97.8	76.8	96.9	91.4	<0.001
Education						0.186
None/Primary	75.9	75.8	75.1	76.9	78.9	
Secondary	19.0	19.1	19.6	18.0	17.0	
Higher	3.1	3.1	3.7	3.4	2.5	
Religious	1.9	2.0	1.6	1.7	1.6	
Support Corporal Punishment	41.4	41.6	39.4	40.2	35.2	<0.001
**Household**						
Wealthiest 40%	35.9	36.1	35.3	33.2	35.2	0.003
Number of Kids	4.4 (0.02)	4.5 (0.02)	4.1 (0.08)	3.8 (0.04)	4.0 (0.15)	<0.001
Relationship to HH Head						<0.001
Child	77.9	81.8	45.0	48.9	4.5	
Other relative	21.7	17.9	54.1	50.0	93.0	
Non-relative	0.4	0.3	0.9	1.1	2.5	
**Community**						
Percent Support CP	40.4 (0.27)	40.5 (0.28)	40.4 (0.75)	38.9 (0.47)	35.4 (1.04)	<0.001
Urban	31.3	31.2	32.8	32.9	26.5	0.002

The statistics in Table 2 confirm that orphans differ from non-orphans in the pooled sample in several key respects. Orphaned children tend to be older, especially double orphans. The average age of non-orphans in the pooled sample is 7.4, lower than maternal (9.1), paternal (9.1), and double (10.0) orphans. Slightly more than half of paternal and double orphans (51.3% and 52.9%, respectively) are girls. Meanwhile, girls are slightly under-represented among maternal orphans (48.5%). Boys may be more likely to strike out independently when a father dies, while girls may be more likely to take this step when a mother passes away. The most notable difference arises in the children’s relationships to household heads. While most non-orphans (81.8%) are the child of the household head, this pattern is reversed among orphaned children. The majority of orphans, even single orphans, are not the offspring of the head of the household in which they are living. Among double orphans, 4.5% are recorded as the child of the household head, suggesting they have been formally or informally adopted.

There are noteworthy dissimilarities in caregivers across orphans and non-orphans. Only 2.2% of the primary caregivers of non-orphans are male. The number of male caregivers is much higher for maternal orphans (23.2%) and double orphans (8.6%). Orphan caregivers also tend to be much older on average than non-orphan caregivers, probably because orphans themselves tend to be older, and there are many orphan caregivers who are grandparents. The average age of non-orphan caregivers is 36.2, while the average age of maternal orphan caregivers is 44.8; paternal orphan caregivers, 42.7, and double orphan caregivers, 49.1. The research shows that male and older caregivers are less likely to use harsh discipline than their younger female counterparts. This may explain why the caregivers of double orphans compared to non-orphan caregivers are less likely to report that violent discipline is necessary to properly raise a child (35.2% versus 41.6%). Differences in attitudes are in the same direction, but less pronounced, for maternal- and paternal-orphan caregivers. Educational differences between caregivers of orphaned and non-orphaned children are small and not statistically significant.

Household differences between orphaned and non-orphaned children are statistically significant. Compared to households with non-orphans, households with orphans are somewhat less likely to be in the top 40 percent in wealth and have fewer children. These differences are strongest for paternal orphans.

Finally, there are significant differences between communities where orphaned and non-orphaned children reside. Non-orphans live in communities where 40.5 percent of caretakers on average support corporal punishment, while double orphans live in communities where support is less common (35.4% on average). Double orphans are also less likely to live in an urban community than non-orphans (26.5% versus 31.2%). The community differences between non-orphans and maternal or paternal orphans are less notable. The range of differences across orphans and non-orphans may explain their varying experiences with harsh discipline. We now turn to multivariable models to see if that is the case.

### 4.4 Logistic regressions

Logistic regression equations estimate the independent association between orphan status and odds of experiencing harsh discipline, after controlling all other relevant factors. In Table 3, Models 1 and 2 show the results for physically violent discipline while Models 3 and 4 show the results for psychologically aggressive discipline. After accounting for country differences (Models 1 and 3), each type of orphan has significantly reduced odds of experiencing physical violence, and maternal and double orphans have reduced odds of experiencing psychological aggression compared with non-orphans. Specifically, maternal orphans, compared to non-orphans, have 34.0% reduced odds of physical violence and 26.8% reduced odds of psychological aggression. Paternal orphans have reduced odds of physical violence (19.1%) but are no different from non-orphans in terms of their experience with psychological aggression. Double orphans also have reduced odds of physically violent and psychologically aggressive discipline (odds reduced by 35.5% and 28.9%, respectively).

**Table 3:** Logistic regression models of harmful punishment.

	Physical Violence				Psychological Aggression			
	(1)		(2)		(3)		(4)	
**Child Characteristics**	OR	95% CI	OR	95% CI	OR	95% CI	OR	95% CI
Orphan Status (Non-orphan)								
Maternal orphan	0.660***	[0.590,0.739]	0.825**	[0.732,0.929]	0.732***	[0.645,0.829]	0.760***	[0.667,0.865]
Paternal orphan	0.809***	[0.758,0.863]	0.911**	[0.850,0.978]	0.969	[0.905,1.038]	0.928*	[0.863,0.997]
Double orphan	0.645***	[0.559,0.744]	0.883	[0.761,1.024]	0.711***	[0.605,0.836]	0.721***	[0.611,0.851]
Age			1.219***	[1.189,1.249]			1.247***	[1.216,1.280]
Age^2^			0.985***	[0.984,0.987]			0.988***	[0.987,0.990]
Female			0.909***	[0.874,0.945]			0.945**	[0.910,0.981]
**Caretaker Characteristics**								
Age			1.009*	[1.002,1.016]			1.013**	[1.005,1.022]
Age^2^			0.999***	[0.999,0.999]			0.999***	[0.999,0.999]
Female			1.473***	[1.329,1.634]			1.354***	[1.214,1.511]
Education (None/Primary)								
Secondary			1.169***	[1.101,1.241]			1.084*	[1.018,1.155]
Higher			0.935	[0.828,1.056]			0.971	[0.868,1.087]
Religious			1.382***	[1.158,1.649]			1.283**	[1.070,1.540]
Support Corporal Punishment			3.119***	[2.963,3.282]			2.224***	[2.114,2.341]
**Household Characteristics**								
Wealthiest 40%			1.060*	[1.007,1.117]			1.002	[0.942,1.066]
Number of Children			1.005	[0.991,1.019]			1.003	[0.988,1.018]
Relationship to Head (Child)								
Non-child relative			0.867***	[0.826,0.909]			0.939*	[0.893,0.987]
Non-relative			0.575***	[0.440,0.752]			0.766*	[0.587,0.998]
**Community Characteristics**								
Proportion Support CP			1.445***	[1.263,1.653]			1.845***	[1.579,2.155]
Urban (Rural)			1.068*	[1.005,1.134]			1.021	[0.949,1.099]
Country Fixed Effects	Yes		Yes		Yes		Yes	
*N*	125,197		125,197		125,197		125,197	

* *p* < 0.05

** *p* < 0.01

*** *p* < 0.001

Exponentiated coefficients; 95% confidence intervals in brackets. Reference group for categorical variables indicated in parentheses.

In Models 2 and 4, we add the full suite of child, caretaker, household, and community characteristics. Adjusting for these covariates explains much of the association between orphan status and physical violence. For double orphans, estimated coefficient is attenuated to non-significance, indicating that their differential exposure to physically violent discipline is explained by these other factors. The results in Model 2 reveal that maternal orphans (OR: 0.825, 95% CI: 0.732–0.929) and paternal orphans (OR: 0.911, 95% CI: 0.850–0.978) still have a modestly lower risk of physically violent discipline than non-orphans.

Comparing Models 3 and 4, we observe that adjusting for covariates does not substantially alter the magnitude nor the significance of the estimated orphan effect for psychologically aggressive discipline. Maternal and double orphans continue to experience about 25% reduced odds of psychological aggression than their peers with both living parents. Interestingly, the estimated effect for paternal orphans grew in magnitude (from 0.969 to 0.928) and reached statistical significance at p < .05 after adjusting for covariates. Psychologically aggressive discipline is more common than violent discipline across the entire sample of children (66.0% versus 75.3%). Apparently, psychologically aggressive discipline cuts more across different household, caregiver, and child characteristics.

Many of the covariates operate similarly for risk of either form of aggressive discipline. Community characteristics significantly impacted odds of harsh punishment. Children living in communities with greater support for corporal punishment had higher odds of experiencing physically violent (OR:1.445, 95% CI: 1.263–1.653) and psychologically aggressive discipline (OR: 1.845, 95% CI: 1.579–2.155). Those living in an urban area had 6.8% greater odds of experiencing physically violent discipline than children living in a rural area, though there were no significant differences with psychological aggression.

In terms of household variables, children in the wealthiest 40% of households had 6.0% greater odds of physically violent discipline than other children, although psychological aggression did not significantly vary by wealth. Number of children in the household had no influence on harsh discipline after controlling for all other factors. Of most interest, children who were not the offspring of the head of their household tended to experience *less* physical violence (13.3% reduced odds when household head was another relative, 42.5% reduced odds when non-relatives) and psychological aggression (6.1% reduced odds when another relative, 23.4% reduced odds when a non-relative). Orphaned children are far more likely to live with relatives other than parents, such as a grandparent or uncle, or with non-relatives, thus reducing their odds of being violently punished relative to non-orphans. The fact that living apart from parents reduces the odds of aggressive discipline is consistent with the interpretation that less harsh punishment signals less investment in a child rather than greater concern for his or her welfare. Another possibility is unobserved heterogeneity not captured in our models.

Odds of harsh discipline varied significantly by caregiver characteristics. Consistent with prior studies, children whose caregivers were women were more likely to experience aggressive discipline (47.3% greater odds of physical violence, 35.4% greater odds of psychological discipline). Maternal and double orphans are considerably more likely to have a male caregiver than other children. In line with previous research, physical violence became slightly less likely with each year of caregiver’s age, which is consistent with the idea that older caregivers age out of violent discipline. However, age had no independent effect on psychological aggression.

In contrast with previous studies, more caregiver education was not consistently associated with reduced odds of aggressive discipline. Compared to caregivers with primary schooling or less, those who completed a secondary education had 16.9% greater odds of reporting physical violence and 8.4% greater odds of reporting psychological aggression. Those who had completed higher education did not significantly differ from those with primary or less schooling, though the point direction is consistent with prior evidence. Caregivers who attended religious schools are most likely to use physically violent and psychologically aggressive discipline.

Children whose caregivers report that physical punishment is necessary to properly bring up a child are also far more likely than others discipline with physical violence (OR: 3.119, 95% CI: 2.963–3.282). Caregivers with this belief also have greater odds of reporting psychological aggression (OR: 2.224, 95% CI: 2.114–2.341). All forms of orphans, especially double orphans, are less likely to have a caregiver who endorses this belief.

Child’s age (measured as age and age-squared to account for the curvilinear relationship between age and risk) was an important predictor of aggressive discipline. The odds of violent discipline is 64% for children at two years of age, increases to 70% by age seven, and then drops off markedly. Orphans are on average older than other children. Including age in the models accounts for this difference and thus reduces the relationship between orphan status and aggressive punishment. Child’s sex was also a significant predictor of discipline. Consistent with previous research, girls were at lower odds of experiencing aggressive discipline in both Models 2 and 4; the gender effect was more pronounced for physical violence (9.1% reduced odds) than psychologically aggressive discipline (5.5% reduced odds).

### 4.5 Supplemental analysis

At first glance, it seems good that orphans are protected from harsh discipline. However, a possible interpretation of these findings is that orphaned children are less likely to receive any form of discipline from their caretakers than non-orphans. If this is the case, it may indicate that orphans are spared from abuse at the cost of being neglected. To tease out this relationship, we estimated the relationship between orphan status and another available indicator of parental investment: school attendance. Despite recent efforts to expand education in LMICs, one in five primary-age children, two in five lower-secondary-age children, and three in five upper-secondary-age children in sub-Saharan Africa were not in school in 2016 [39]. The costs of education include both school fees and diminished household labor provided by children [40]. These costs make education a significant parental investment in this region. If orphaned children receive less investment than their peers, we would expect them to have a lower prevalence of school attendance.

Results of this supplementary analysis are displayed in Table 4. These models were restricted to children aged 5–14, since younger children were not eligible for formal schooling in most sample countries. That is why the sample size (N = 90,604) is somewhat smaller than the main analyses. In the bivariate model controlling only country fixed effects (Model 1), there is heterogeneity among types of orphans. Maternal orphans and double orphans are just as likely to attend school as their peers, while paternal orphans have somewhat greater odds (OR: 1.170, 95% CI: 1.076–1.271). Adjusting for child, household, and caretaker covariates changes these estimates substantially. In Model 2, we observe that maternal and paternal did not significantly differ from non-orphans after adjusting for other factors. Double orphans experienced the largest difference, with 39.6% reduced odds of school attendance compared to their peers with living parents. These results, which are consistent with previous research [41], mimic the pattern of significant differences for orphans observed in our analyses of violent and psychologically-aggressive discipline. These findings give additional weight to our interpretation that lower investment explains the reduced risk of harsh discipline among orphaned children.

**Table 4:** Logistic regression models of school attendance.

	(1)		(2)	
Child Characteristics	OR	95% CI	OR	95% CI
Orphan Status (Non-orphan)				
Maternal orphan	1.008	[0.875,1.161]	0.861	[0.733,1.011]
Paternal orphan	1.170***	[1.076,1.271]	0.969	[0.885,1.062]
Double orphan	0.843	[0.697,1.020]	0.604***	[0.493,0.740]
Age			4.075***	[3.811,4.358]
Age^2^			0.937***	[0.933,0.940]
Female			0.825***	[0.783,0.868]
Relationship to Head (Child)				
Non-child relative			1.000	[0.932,1.073]
Non-relative			0.210***	[0.146,0.302]
**Caretaker Characteristics**				
Age			1.013*	[1.003,1.024]
Age^2^			0.999	[0.999,1.000]
Female			1.240**	[1.073,1.433]
Education (None/Primary)				
Secondary			3.524***	[3.171,3.916]
Higher			4.572***	[3.460,6.042]
Religious			1.826***	[1.464,2.278]
Support Corporal Punishment			1.021	[0.963,1.082]
**Household Characteristics**				
Wealthiest 40%			2.594***	[2.386,2.821]
Number of Children			0.977**	[0.962,0.993]
Relationship to Head (Child)				
Non-child relative			1.000	[0.932,1.073]
Non-relative			0.210***	[0.146,0.302]
**Community Characteristics**				
Proportion Support CP			1.223	[0.999,1.497]
Urban (Rural)			1.496***	[1.357,1.649]
Country Fixed Effects	Yes		Yes	
*N*	90,604		90,604	

Exponentiated coefficients; 95% confidence intervals in brackets. Reference group for categorical variables indicated in parentheses.

* *p* < 0.05

** *p* < 0.01

*** *p* < 0.001

## 5. Discussion

Our analyses reveal that orphans are *less* likely to endure physically violent or psychologically aggressive discipline than non-orphans in sub-Saharan Africa. Part of this association is explained by the fact that orphaned children in our sample are more likely to live in countries where harsh discipline is less common overall. However, there are also stark disciplinary differences between orphans and non-orphans within many countries. Both between- and within-country differences contribute to the total disciplinary differences across orphan status in the pooled sample.

In the bivariate models, double orphans were least at risk of harsh discipline. This difference became statistically insignificant for physically violent discipline when we added control variables to the models, but it remained statistically significant for psychologically aggressive discipline. Paternal orphans most closely resembled non-orphans in terms of both physical and psychological violence. This suggests that having a deceased mother is a key factor producing differences between orphans and non-orphans. The finding also aligns with the fact that male caretakers are less likely to use aggressive forms of discipline, and most paternal orphans receive primary care from their living mother. Although differences in the risk of harsh discipline are reduced when we control for country, community, household, caretaker, and child characteristics; for the most part, they are not fully explained by these controls.

We propose two possible explanations for our surprising findings. One explanation is methodological—that our models, while extensive, are still missing some important differences across orphans and other children. For example, it is possible that the caretakers of orphans (whether a surviving parent or someone else) are on average more empathetic than other caretakers. They may be especially gentle with orphans to avoid adding to these children’s trauma. This is consistent with compensatory theories of parenting [42], which note that caretaker stress sometimes translates into greater protections for children. Caretaker empathy is an example of something we could not measure in our models that might explain less harsh punishment for orphans.

A very different explanation relates to the community meaning ascribed to aggressive discipline. In communities where corporal punishment is normative, less harsh discipline could indicate less caretaker investment in a child. At least one element of the multivariable model suggests this could be the case. When a child is the offspring of the head of the household, his or her risk of aggressive discipline is dramatically *increased*. This suggests that more aggressive discipline is associated with more concern for children rather than the other way around.

To further explore our two competing explanations, we conducted a supplemental analysis considering orphans versus non-orphans’ school enrollment. The patterns uncovered in that analysis are similar to our findings for harsh discipline. Double orphans were less likely than non-orphans to be enrolled in school, which is a clear a sign of lower caregiver investment. All other factors controlled, paternal orphans were just as likely to be enrolled in school as children whose parents were both alive. This suggests that the mothers of single orphans make significant investments in their children’s well-being. Overall, our similar findings for discipline and school enrollment are most consistent with the interpretation that lower caregiver investment in orphans explains these children’s lower odds of experiencing aggressive discipline. Additional evidence, particularly from qualitative studies or household surveys that collect discipline data from multiple children in a home, will be important to definitively determine why orphans in general experience less harsh discipline than other children.

In terms of predicting the risk of harsh punishment in general, community norms supporting corporal punishment are associated with a greater risk of harsh punishment, as is caretakers’ individual support for corporal punishment. Caretaker age, gender, and education are all associated with harsh discipline. Children supervised by younger men and caretakers with a religious educational experience are more likely to experience both physically and psychologically aggressive discipline, as are children cared for by a biological parent. Boys in the middle-age range are most at risk.

Some of the ill effects of violent discipline arise from children feeling rejected by their caretakers when they are physically disciplined [34]. Community norms supporting aggressive discipline can mediate this harm by providing an alternative interpretation of violent discipline, one that is not associated with rejection. Notwithstanding the subjective meaning assigned to aggressive discipline, the global community agrees on the need to curb such practices. Aggressive discipline has overall negative effects, even in settings where the practices are highly normative [34]. It is therefore heartening to know that orphans, who are disproportionately at risk of many harms, are less likely to experience aggressive discipline.

It is important to note that our study has some limitations. By relying on household survey data, this study does not consider orphans who are homeless or live in institutions. These orphans may be more likely to experience physical violence and psychological aggression than orphans who are taken into a home. Additionally, our data on discipline is self-reported by the child’s caretaker. It is possible that favorability bias may have caused underreporting of certain types of discipline, particularly the more severe forms of physical violence. However, we have no reason to believe that any underreporting would differ systematically between orphaned and non-orphaned children. Also, the fact that over 40 percent of caretakers in our pooled sample expressed that physical punishment is necessary to properly bring up a child indicates that this type of discipline is likely normative in most of the countries in our study. Although the scale used to measure child discipline in MICS has been validated in international settings, it was not thoroughly validated in every country or population represented in this analysis. Our analysis is also limited to the 14 countries that had MICS data. We thus suggest caution in generalizing these results to other types of orphans or orphans in other countries.

## Supporting information

S1 Table Child discipline module in multiple indicator cluster survey. (DOCX)
