# Supplementary material for: Disciplinary practices among orphaned children in Sub-Saharan Africa
Source: PLoS One. 2021 Feb 4;16(2):e0246578. doi: 10.1371/journal.pone.0246578 (PMC7861390; doi:10.1371/journal.pone.0246578)
Supplement: S1 Table — (DOCX) [file pone.0246578.s001.docx]

| S1 Table. Child Discipline Module in Multiple Indicator Cluster Survey | |
| --- | --- |
| Question Preamble: Adults use certain ways to teach children the right behavior or to address a behavior problem. I will read various methods that are used. Please tell me if you or anyone else in your household has used this method with (child’s name) in the past month. | |
|  | |
| Non-violent | - Took away privileges, forbade something (child’s name) liked or did not allow him/her to leave the house. |
|  | - Explained why (child’s name)’s behavior was wrong. |
|  | - Gave him/her something else to do. |
|  | |
|  | |
| Physically Violent | - Shook him/her. |
|  | - Spanked, hit, or slapped him/her on the bottom with bare hand. |
|  | - Hit him/her on the bottom or elsewhere on the body with something like a belt, hairbrush, stick, or other hard object. |
|  | - Hit or slapped him/her on the face, head or ears. |
|  | - Hit or slapped him/her on the hand, arm or leg. |
|  | - Beat him/her up, that is hit him/her over and over as hard as one could. |
|  | |
|  | |
| Psychologically Aggressive | - Shouted, yelled at or screamed at him/her. |
|  | - Called him/her dumb, lazy, or another name like that. |
|  | |
|  | |
| Follow Up Question: Do you believe that in order to bring up, raise, or educate a child properly, the child needs to be physically punished? | |
| Note: Caretakers check yes/no to each specific disciplinary action used with the selected child. These actions are coded as non-violent, physically violent, or psychologically aggressive according to the typology used by UNICEF. | |
